# Taylor spatial frame-software-controlled fixator for deformity correction-the early Indian experience

**DOI:** 10.4103/0019-5413.32052

**Published:** 2007

**Authors:** Milind Chaudhary

**Affiliations:** Govt Medical College, Akola, CFIT India, Jaslok Hospital, Mumbai

**Keywords:** Deformity correction, Ilizarov, limb lengthening, taylor spatial frame fixator

## Abstract

**Background:**

Complex deformity correction and fracture treatment with the Ilizarov method needs extensive preoperative analysis and laborious postoperative fixator alterations, which are error-prone. We report our initial experience in treating the first 22 patients having fractures and complex deformities and shortening with software-controlled Taylor spatial frame (TSF) external fixator, for its ease of use and accuracy in achieving fracture reduction and complex deformity correction.

**Settings and Design:**

The struts of the TSF fixator have multiplane hinges at both ends and the six struts allow correction in all six axes. Hence the same struts act to correct either angulation or translation or rotation. With a single construct assembled during surgery all the desired axis corrections can be performed without a change of the montage as is needed with the Ilizarov fixator.

**Materials and Methods:**

Twenty-seven limb segments were operated with the TSF fixator. There were 23 tibiae, two femora, one knee joint and one ankle joint. Seven patients had comminuted fractures. Ten patients who had 13 deformed segments achieved full correction. Eight patients had lengthening in 10 tibiae. (Five of these also had simultaneous correction of deformities). One patient each had correction of knee and ankle deformities. Accurate reduction of fractures and correction of deformities and length could be achieved in all of our patients with minimum postoperative fixator alterations as compared to the Ilizarov system. The X-ray visualization of the osteotomy or lengthening site due to the six crossing struts and added bulk of the fixator rings which made positioning in bed and walking slightly more difficult as compared to the Ilizarov fixator.

**Conclusions:**

The TSF external fixator allows accurate fracture reduction and deformity correction without tedious analysis and postoperative frame alterations. The high cost of the fixator is a deterrent. The need for an internet connection and special X-rays to operate the fixator add to its complexity.

The Ilizarov fixator is best known for limb lengthening and deformity corrections. It has a well-defined role for the treatment of complex nonunions with infection and bone loss. It is also well established in the treatment of compound fractures.

While application of the Ilizarov fixator itself is not difficult for simpler cases, the postoperative management in terms of modification of the apparatus for secondary or sequential corrections can be time-consuming and fraught with error. The analysis of the oblique plane and combined angular and rotational deformities can pose significant challenges to surgeons who are less mathematically or mechanically inclined.

The Taylor spatial frame (TSF) fixator was invented by Dr. J. Charles Taylor of Memphis USA (1990) which was designed to overcome these difficulties of the use of the Ilizarov fixator.^1^ It is a software-driven hexapod fixator made with circular rings with six struts instead of the three or four used with the Ilizarov. It is based on the science of Projective Geometry and is the offshoot of the Chasles Theorem.^2^ It is based on a principle similar to that used in Aircraft Simulators.

We hereby report our experience of “Taylor's Spatial Frame” in a series of 27 limbs in 22 patients.

## MATERIALS AND METHODS

Twenty-four patients were operated with the TSF fixator from Oct 2001 to June 2006, of whom 22 are included in the study. Their ages ranged from 13 years to 56 years. Twenty-seven limb segments were operated. There were 23 tibiae, two femora, one knee joint and one ankle joint [Table 1].

**Table 1:** Patients

Sr No.	Name	Age	Sex	Bone	Side	Diagnosis	Treatment	Result	Comments
1	BD	56	M	Tibia	R	# upper tibia	Cl Redn Ex fixation	Union 14 weeks	No residual deformity and compl
2	PJ	31	F	Tibia	L	# upper tibia	Cl Redn Ex fixation	Union 12 weeks	Polio. No residual deformity and compl
3	AJ	55	M	Tibia	R	# lower tibia	Cl Redn Ex fixation	Union 16 weeks	Needed a change of struts in the end for better visualization
4	GN	24	M	Tibia	L	# comm. M3 tibia	Cl Redn Ex fixation	20 weeks union	Assoc. Femoral # Rx with Ilizarov fixator
5	MK	58	M	Tibia	R	# lower 3rd	Cl Redn Ex	22 weeks union	Needed bone grafting and Pin exchange
6	JK	32	M	Tibia	L	# m3 tibia	Cl Redn Ex fixation	Union	Assoc bilat # Colles
7	RV	33	M	Femur	R	U3#	Cl Redn Ex fixation	Union 24 weeks	Assoc Sciatic N Palsy. Needed Neurolysis, nerve recovered. Mild residual deformities.
8	VM	19	F	Tibia	R	Growth arrest+ Varus recurv and IR deformity.	Lengthening and Deformity correction	3.5 cm lengthening. Full correction of deformity	Struts changed to Ilizarov due to difficulty in implementing program
9	AB	23	M	Tibia	L	Growth arrest + Varus-Procurvatum	Lengthening and Deformity correction	4 cm lengthening	Bone cyst in U tibia caused Growth arrest. Corticotomy distal-caused minimal medial translation
10	PM	36	M	Tibia	R	Malunion-Varus, Procurvatum, medial and anterior translation	Lengthening and Deformity Correction	3 cm lengthening	Accurate correction of length through a corticotomy done through abnormal soft tissues
11	YSKK	39	M	Tibia	L	Malunion-varus, procurvatum, shortening	Lengthening and Deformity Correction	2.8 cm lengthening	Developed procurvatum deformity during treatment. Ran program to correct.
12	CU	32	M	Tibia	R	Malunion-varus, procurvatum, anterior translation	Lengthening and Deformity correction foll rule 2	Achieved 3 cm of length	Injury due to Gunshot wounds. Residual shortening of 12 mm due to premature consolidation
13	SJ	21	M	Femur and Tibia	L	Malunion-femur procurvatum 85°, Tibia recurvatum 60°	Deformity correction	Full correction of both deformities	Needed no lengthening
14	TB	26	M	Tibia	L	Valgus Deformity	Deformity correction	Done acutely	Full correction. Also had Femoral valgus to be corrected later
15	VS	33	F	Tibia	Bilat	Valgus def 7°	Deformity correction	Gradual correction	Lat Popl. Nerve Decompression done on one side. Also had Femoral valgus corrected with Monorail fixator
16	UP	11	M	Tibia	Bilat	Varus-Procurvatum	Deformity correction	Acute correction	Chondro Metaphyseal dysplasia. Full correction
17	SC	21	M	Tibia	L	Varus	Deformity correction	Acute correction	Full correction achieved
18	MH	18	F	Tibia	R	Cong. PM Bowing	Lengthening and deformity correction	3 cm length with deformity correction	Full correction and equalization of Limb Lengths
19	GK	31	M	Tibia	Bilat	Constitutional Short Stature	Lengthening	6 cm length achieved	Mild residual procurvatum on one side due to instability
20	SS	21	M	Tibia	Bilat	Constitutional Short Stature	Lengthening	6 cm length	Equal lengths with no residual deformity
21	MP	13	F	Ankle	R	Polio with severe Equinus ankle joint	Gradual correction of equinus	Overcorrection into 5° dorsiflexion achieved	No overdistraction of ankle or crushing of cartilage. Splintage postoperatively for maintainence
22	NR	29	M	Knee Joint	R	Chronic Traumatic Dislocation of knee	Gradual reduction and arthrodesis	Sound fusion	Assoc femur and tibia fractures. Protected in fixator. Femur went on to Nonunion later

Eight patients underwent lengthening with or without deformity correction in 10 tibiae. The length gained ranged from 2.5 cm to 6.5 cm. Three patients underwent only lengthening in five tibiae. Of these three patients, one had Congenital Postero-Medial Bowing of the tibia and the other two had constitutional short stature. Two patients had growth arrest with shortening and deformities in two tibiae and underwent tibial lengthening and deformity correction. Three patients with posttraumatic tibial malunion underwent lengthening with deformity correction in three tibiae. All aforesaid tibial malunions were in varus, procurvatum and anterior translation deformities along with shortening of 2.8-4 cm.

Ten patients with 13 limb deformities (12 tibiae, one femur) underwent deformity correction with or without lengthening. There were seven tibial varus-procurvatum deformities, one tibial varus, one tibial varus-recurvatum deformity and three tibial valgus deformities, which underwent correction. Five patients were common to the lengthening as well as the deformity correction group.

One patient who had an ipsilateral femoral procurvatum of 88° and tibial recurvatum deformity of 60° [Figures 1a–h] achieved full correction with the help of the TSF fixator. He had a malunion of the femur in childhood which was untreated and neglected and hence resulted in a compensatory deformity in the upper tibia. He had no shortening and hence underwent pure deformity correction with a gradual angulation-translation maneuver.

**Figure 1:**
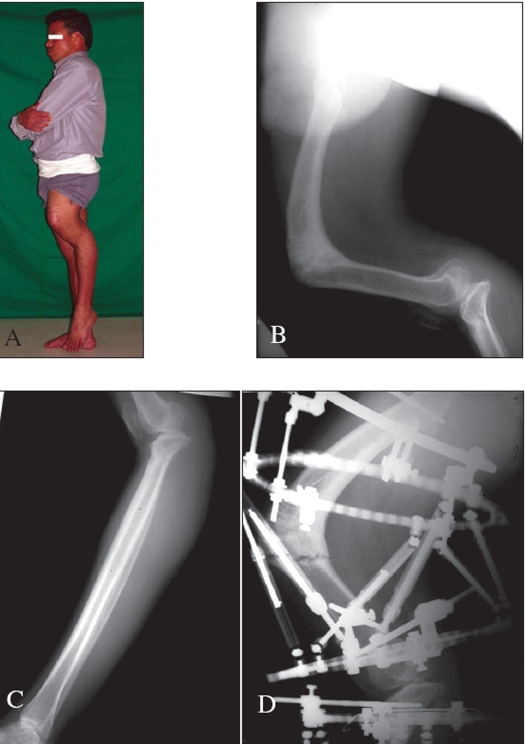
A) Severe deformities in sagittal plane after childhod injury and multiple surgeries. B) Severe procurvatum deformity in middle femur more than 80 degrees. C) 60 degrees of recurvatum in the upper tibia very close to the articular surface. D) TSF fixator applied with 6 criss-crossing telescoping graduated struts with a percutaneous osteotomy

**Figure 1:**
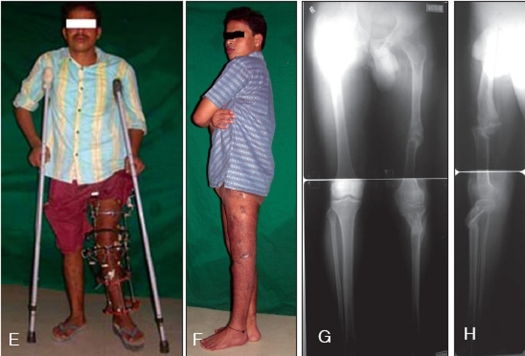
E) The taylor spatial frame fixator permits walking and mobilization when due care is taken. F) Clinical result showing full correction of femoral procurvatum and tibial recurvatum. G) Full length AP X-ray showing complete correction of deformity and mechanical axis passing through the center of the knee. H) Full length lateral X-ray showing the mechanical axis passing through the center of the knee joint even though there is a mild translation of the distal femur anteriorly and distal tibia posteriorly. See text for explanation

One patient suffering from postpolio residual paralysis had a 60° ankle joint equinus contracture. The equinus was not correctible on knee flexion. The TSF frame was used to create a virtual centre of rotation at the level of the ankle joint instant centre. Correction was achieved with gradual soft tissue distraction.

One patient with monomelic polytrauma had a fracture shaft femur and shaft tibia which were plated, but also had a dislocation of the knee which was neglected. It presented with varus, posterior translation and overriding 5 months after the trauma. The TSF fixator spanned the knee and was extended above into the femur and below into the tibia with Ilizarov rings to protect the plated shaft fractures which had not healed. The TSF fixator achieved gradual reduction of the neglected dislocation.

Seven patients (tibia=six, femur=one) had complex or comminuted fractures. The six tibial fractures were two each in the upper, middle and lower third of the tibia. In all the patients two TSF rings closest to the fracture site and Illizarov rings elsewhere were used. A four-week old compound subtrochanteric fracture following gunshot injury presented with complete sciatic nerve palsy and a large wound over the posterior thigh with embedded pellets. The TSF fixator was used to reduce the fracture and allow wound debridement as well as mobilization of the patient. When the posterior thigh wound healed, a sciatic neurolysis was done between the rings of the TSF fixator with the patient in prone position. The nerve was in continuity, but enmeshed in fibrous tissue. It was freed completely and started showing early recovery in 10 weeks and nearly full function in 24 weeks.

**Figure 2:**
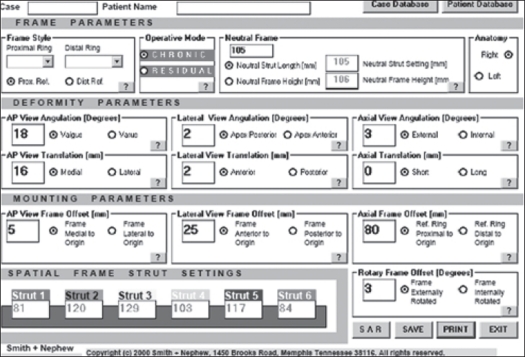
Shows the software interface to be used in the chronic mode

Till 2003 we used the software in the Chronic and Residual Mode [Figure 2] in 10 patients and since then we have used the web-based Total Residual^1^ software in the remaining 12 patients.

Fixation to bone was achieved with wires in the fracture group and wire-half pin hybrid in the rest. These were fixed to the aluminum rings by wire fixation bolts and Rancho cubes, clamps etc. The aluminum rings are thicker than Ilizarov rings but are radiolucent. The control over bony fragments for deformity correction, lengthening or fracture reduction is done with the help of software. X-ray measurements^3^ in the AP and lateral plane are taken which measure the angular, rotational and length deviations.

Special measurements which allow the software to get oriented to the bone fragment position vis-á-vis the size and orientation of the rings, are taken with the help of a translucent grid on the X-rays and fed into the software. The ring and strut sizes are also fed in as are the desired correction parameters. A note is taken of any structure at risk like the peroneal nerve in the region of neck of fibula, so as not to stretch it. The software then outputs a program, which guides the surgeon and patient to turn the struts at specific intervals and amounts to achieve the desired result. Patients could follow instructions very easily as the struts were color-coded with tags.

Surgery was conducted under spinal or general anesthesia depending on the patients' preference. All patients had full segment X-rays before and during treatment. During the postoperative phase, X-rays were made perfectly parallel to the reference ring, exactly orthogonal to the bone and had to include the entire width of the ring to ensure that the virtual centre of the ring could be calculated accurately for measuring the frame offsets.

Length measurements were made using magnification markers in the interim and with scanograms towards the beginning and end of treatment. The minor secondary deformities that arose during lengthening were also corrected. All the basic principles of frame application as for the Ilizarov fixator were applied to preserve joint range of motion. Most of the applications were in the tibia where the foot was not fixed and the ankle was kept free. Weight-bearing ambulation was ensured in every patient. The principles of preserving the knee range of motion are the same for either the Ilizarov or the Taylor spatial frame fixators.

## RESULTS

All patients achieved lengthening without any major complications. One tibia developed a 7° residual procurvatum in the consolidation phase of lengthening which could have been avoided had the patient agreed to a pin-exchange. One patient with varus-procurvatum malunion with shortening could not achieve full length correction due to premature consolidation. The deformities were fully corrected but the length fell short by 12 mm. He refused a repeat corticotomy.

The deformity correction patients achieved full correction of the deformities that ranged from 14° to 88° without any residual deformity of more than 1°. In the illustrated case [Figure 1h], the apparent anterior translation of the tibia is due to the deformity correction being done with Rule 2 of deformity correction principles.^4^ It means when the deformity is corrected by placing the hinges exactly at the “CORA” (centre of rotation of angulation), but the osteotomy itself is performed at a level different from the true apex of the deformity, full correction of the overall axis will occur, albeit with translation at the osteotomy site. If noticed carefully, the mechanical axis of the limb is passing exactly through the centre of the knee. Rule 2 was employed and the osteotomy was performed distal to the level of the sub-articular deformity. There was not enough space for the upper tibial ring to have gone a little closer to the joint line due to the presence of the tilted lower femoral ring due to its severe deformity. Thus the osteotomy got shifted a little distally and necessitated a posterior translation of the distal fragment to achieve correction. Any more posterior translation of the distal tibial fragment would have caused a lack of contact of the bony fragments. Hence it was decided to accept the small resultant anterior translation of the tibial mechanical axis as it did not exceed 12 mm. However, it may be clearly observed that the overall mechanical axis is completely corrected and passes exactly through the centre of the knee joint. This has been possible due to the small anterior translation of the distal femoral fragment. The femoral and tibial translations have the opposite effects on the position of the mechanical axis at the level of the knee joint.

All fractures united. Case no. 7 with Compound subtrochanteric femur fracture healed with 5° varus and 11° procurvatum. He also had a sciatic nerve palsy for which a neurolysis was done. The nerve started recovery after 10 weeks and recovered completely by six months. Case no. 5 with a comminuted lower third tibia needed an iliac crest bone grafting and pin exchange to achieve union.

With TSF fixator it was possible to fix the fracture in an emergency without worrying about the reduction. We could run the software program later to achieve a perfect reduction of the fracture fragments gradually and without pain.

Case 22 with knee joint dislocation underwent a relocation and then was converted to a sound knee arthrodesis without deformity. Case 21 with ankle joint equinus contracture was corrected completely to an overcorrection of 5° and has not recurred three years post frame removal. There were no major pin track infections or severe limb pain during treatment.

## DISCUSSION

The Ilizarov fixator is accepted as the gold standard for correction of complex deformities. A thorough understanding of the complex principles of deformity correction is necessary to analyze and accurately treat deformities. This would include some basic and applied trigonometry. The amount of time required to analyze a complex oblique plane deformity, can be daunting to the casual surgeon.

The steps are: measure the deformity in the antero-posterior and lateral X-rays. With the graphical method of analysis on paper, an outline of the Ilizarov ring size to be used is drawn and the cross-section of the bone is drawn to simulate a transverse section of the limb at the site of the maximum deformity. The antero-posterior deformity is drawn as a line on the abscissa (X-axis) and the lateral plane deformity is drawn as a line on the ordinate (Y-axis). A rectangle is completed, the length of the diagonal of which gives the true magnitude of the deformity. The angle of inclination of this diagonal from the abscissa gives the plane of the deformity.

Hinges are placed on a line which is Orthogonal to this diagonal as it meets the ring outline. The diagonal itself is extended in the concavity of the deformity to give the position of the motor rod. Then, a preconstruction of the frame is made based on this graphical analysis, usually with the patient examined under a C-Arm before surgery. This process can take several hours and can be very confusing to the surgeon

In the treatment of compound or comminuted fractures or lengthening, secondary deformities usually develop during treatment. These need to be corrected by changing the Ilizarov frame montage.^5^ This can be time-consuming and requires a mechanical aptitude and is prone to error. The most difficult of these constructs are the rotational and translation\ constructs, especially when combined with an angular deformity.

The TSF fixator has all the advantages of the Ilizarov fixator and overcomes some of its above mentioned difficulties. The surgeon is greatly benefited by its ease of use^6^ as well as the modern and high-tech interface. Other parameters remaining the same, the TSF fixator makes preoperative planning and postoperative alterations of the fixator extremely simple. The software package takes away the tedium of extensive calculations needed for preoperative planning. It requires 13 simple measurements from standard AP and lateral X-rays. The software does the calculations necessary to analyze the oblique plane or get the fracture to reduce accurately.

The six struts have multiplane hinges built in at both their ends which can serve any desired function: namely, lengthening, shortening, angular, translational or rotational correction. Once the fixator is applied, all of these complex alterations can take place without any further labor and changes to the fixator. This allows great ease of use for the surgeon and ensures accurate reduction.

There are a few disadvantages, however. The aluminum tabbed rings are bulkier (albeit lighter) than the Ilizarov ones. The six struts in a criss-crossing pattern frequently obliterate the view of the regenerate bone or fracture site. The decision to remove the fixator after judging for full consolidation or healing became somewhat difficult. We solved the problem by replacing the TSF fixator struts with those of the Ilizarov system to better visualize all sectors of the regenerate or the osteotomy site.

The computer software is only able to produce accurate results if the input is precise. There is a possibility that a single wrong measurement may make the bone turn in the wrong direction. It has happened twice to the author. The TSF fixator system is costly and requires a computer system or an internet connection for surgical planning and postsurgical alteration. The system and its nomenclature need familiarity by using it frequently to afford accuracy.

An experienced surgeon using the Ilizarov system for many years, with a sound knowledge of the principles of deformity correction can certainly achieve results that are very accurate. This is borne out in the author's experience.

However, our results using the TSF fixator in 27 segments over the last five years have yielded us very accurate and gratifying results. The fixator is a definite advance over the older Ilizarov system and it is easy to use.

## CONCLUSION

The TSF fixator is a computer software-controlled circular external fixator using six struts, allowing correction of bony deformities, fractures and limb lengthening with great accuracy and ease of use. The ease of use is evident in the preoperative analysis as well as postoperative fixator alterations.
